# The circadian clock mediates the response to oxidative stress in a cone photoreceptor‒like (661W) cell line via regulation of glutathione peroxidase activity

**DOI:** 10.12688/f1000research.125133.2

**Published:** 2022-11-21

**Authors:** Kenkichi Baba, Ting-Chung Suen, Varunika Goyal, Adam Stowie, Alec Davidson, Jason DeBruyne, Gianluca Tosini

**Affiliations:** 1 Department of Pharmacology and Toxicology, Morehouse School of Medicine, Atlanta, Georgia, 30310, USA; 2 Neuroscience Institute, Morehouse School of Medicine, Atlanta, Georgia, 30310, USA; 3 Department of Neurobiology, Morehouse School of Medicine, Atlanta, Georgia, 30310, USA

**Keywords:** Circadian rhythm, Bmal1, Photoreceptors, 661W cells, Oxidative stress, Antioxidant, Nrf2, GPX

## Abstract

**Background**: The mammalian retina contains an autonomous circadian clock that controls many physiological functions within this tissue. Our previous studies have indicated that disruption of this circadian clock by removing *Bmal1* from the retina affects the visual function, retinal circuitry, and cone photoreceptor viability during aging. In the present study, we employed a mouse-derived cone photoreceptor‒like cell, 661W, to investigate which molecular mechanisms of the circadian clock may modulate cone photoreceptor viability during aging.

**Methods**: *Bmal1* knockout (BKO) cells were generated from 661W cells using the CRISPR/Cas9 gene editing tool. Deletion of *Bmal1* from 661W was verified by western blot and monitoring *Per2-luc* bioluminescence circadian rhythms. To investigate the effect of *Bmal1* removal on an oxidative stress challenge, cells were treated with hydrogen peroxide (H _2_O _2_,1 mM) for two hours and then cell viability was assessed. Cells were also cultured and harvested for gene expression analysis and antioxidant assay.

**Results**: Our data indicated that 661W cells contain a functional circadian clock that mediates the response to an oxidative stress challenge *in vitro* and that such a response is no longer present in the BKO cell. We also hypothesized that the effect was due to the circadian regulation of the intracellular antioxidant defense mechanism. Our results revealed that in 661W cells, the antioxidant defense mechanism showed time dependent variation , whereas in BKO cells, there was an overall reduction in this antioxidant defense mechanism, and it no longer showed time dependent variation.

**Conclusions**: Our work supported the notion that the presence of a functional circadian clock and its ability to modulate the response to an oxidative stress is the underlying mechanism that may protect cones during aging.

## Introduction

The presence of a retinal circadian clock in mammals was demonstrated in the 1990s, ^1^^,^^2^ and many studies have shown that retinal circadian clocks control many physiological functions within the retinal tissue. ^3^ Additional studies using mice in which clock genes have been deleted have reported that the disruption of retinal circadian clocks has a profound effect on the retina. ^4^^–^^7^ In the retina, clock genes are expressed in the photoreceptors and inner nuclear and ganglion cell layers. ^8^^–^^11^ In the photoreceptor layer, only the cone photoreceptors seem to express clock genes. ^9^ Consistent with these observations, a series of recent studies using a retina-specific *Bmal1* knockout (KO; *Chx10 ^Cre^; Bmal1 ^fl/fl^*) mouse have reported that removal of the *Bmal1* gene abolished the circadian rhythm of the photic (cone) electroretinogram, ^4^ altered the spectral identity, ^12^ and the viability of the cone photoreceptors during aging. ^13^ Hence, disruption of the circadian clock in the cones affected many biological functions of these cells. ^14^

Photoreceptors, especially cones, have a high metabolic rate ^15^^,^^16^ and contain more mitochondria than rods. ^17^^,^^18^ Consequentially, cones produce a high level of reactive oxygen species (ROS). ROS are by-products of mitochondrial aerobic metabolism and an accumulation of ROS during aging is natural and inevitable. ^19^^,^^20^ Elevated intracellular levels of ROS cause oxidation and damage to lipids, proteins, nucleotides, and mitochondria. ^21^^,^^22^ The daily activity of an organism is closely connected with ROS production, and rhythmic ROS production/cellular oxidation is reported in many organs. ^23^^–^^25^ Furthermore, the removal of *Bmal1* increases ROS levels in a variety of mammalian organs. ^26^^–^^30^

Hence, it is possible to speculate that the reduction in cone viability observed in our previous study in mice lacking *Bmal1* ^14^ may have been due to a dysfunction in the circadian regulation of the cells’ antioxidant defenses. Unfortunately, in the mouse retina, cones comprise only 2% to 3% of the total photoreceptors; thus, performing *in vivo* mechanistic studies of these cells is challenging.

Recent studies have reported that 661W cells, which are a murine cone-like photoreceptor cell line developed by Tan *et al*., ^31^ are useful for investigating the fundamental aspects of cone biology *in vitro*. ^31^^,^^32^ Using these cells, several studies have provided important insights into the molecular mechanisms regulating photoreceptor metabolism ^33^^–^^35^ and death following exposure to oxidative stress. ^36^^,^^37^

The aim of the present study was first to investigate whether 661W cells contain a functional circadian clock and then to explore whether the cells would be useful for investigating the role of the circadian clock in the modulation of their response to oxidative stress.

## Methods

### Cell culture

661W cells (RRID:CVCL_6240) were grown in Dulbecco’s Modified Eagle Medium (DMEM; Gibco, Life Technologies, Carlsbad, CA, USA) supplemented with 5% fetal bovine serum (Gibco) and 1% penicillin/streptomycin at 37°C in a 5% CO _2_ humidified atmosphere. ^37^ Cells were seeded in 35 mm or 100 mm dishes, 96-well plates (Corning, Inc., Corning, NY, USA), or chamber slides (Lab-Tek, Inc., Grand Rapids, MI, USA) at a concentration of 1 × 10 ^4^ to 5 × 10 ^5^ cells in a volume of 0.2 to 5 mL of media and grown to approximately 50% to 90% confluence, depending on the experimental protocol. The number of cells was determined using the cell counter (Bio-Rad TC20 Automated Cell Counter) and the cell survival rate was calculated from the number of living cells out of total cell counts.

### Establishment of *Period2-luciferase* ( *Per2-luc)* 661W cell line and real-time bioluminescence monitoring

*Per2-luc*, a plasmid that expresses the luciferase gene driven by the Per2 promoter, was used. ^38^ Briefly, 1 μg of *Per2-luc* and 0.1 μg of pcDNA3 (which expresses the neomycin-resistant gene driven by the cytomegalovirus enhancer-promoter) was transfected by lipofectamine 2000 (Invitrogen, Waltham, MA, USA) into the 661W cells. The cultured 661W cells were then treated with a final concentration of 400 μg/mL geneticin and geneticin-resistant colonies were selected for bioluminescence verification. Cell lines that express luciferase activity in a circadian manner were identified by the bioluminescence emitted from 661W cells. *Per2-luc-*transfected 661W cells were cultured in DMEM containing 0.1 mM D-luciferase K salt (Molecular Imaging Products Co., Bend, OR, USA), and the bioluminescence emitted from the cells was monitored with either Lumicycle (Actimetrics, Wilmette, IL, USA; see details in Baba *et al*. ^39^) or a charged-coupled device (CCD) camera (Stanford Photonics, Palo Alto, CA, USA; XR Mega 10Z; see details in Evans *et al*. ^40^ and Baba *et al*. ^39^). Briefly, the bioluminescence images of cultured 661W cells were obtained by a Zeiss AxioObserver Z1 microscope (Zeiss, Oberkochen, Germany) with an ×10 Fluar objective lens (Zeiss), Marzhauzer scanning stage with LUDL Mac 5000 controller, and a Stanford Photonics (Palo Alto, CA, USA) XR Mega 10Z cooled intensified CCD camera in a custom-built light-tight chamber maintained at 37°C. Bioluminescence rhythms recorded by Lumicycle were analyzed by Lumicycle analysis software (Actimetrics), and a peak of the *Per2-luc* rhythm was identified by Origin software (OriginLab Corp., Northampton, MA, USA; see details in Baba *et al*. ^41^). Briefly, bioluminescence recordings obtained from Lumicycle were detrended by a 24-hour moving average subtraction method and smoothed by a 2-hour moving average. The circadian peak phase was determined as the highest point of the curve picked by Origin software. *Per2-luc* bioluminescence emitted from 661W cells was captured by a CCD camera every 30 minutes for three days. The intensity of the bioluminescence from consecutive captured images was analyzed with ImageJ software (National Institutes of Health [NIH], Bethesda, MD, USA).

### Establishment of *Bmal1* KO cell lines using the CRISPR/Cas9 system

A construct to knock out *Bmal1* was purchased from Santa Cruz Biotechnology, Inc. (Dallas, TX, USA; BMAL1 CRISPR/Cas9 KO Plasmid, Santa Cruz sc-419206). The BMAL1 CRISPR/Cas9 KO plasmid is a pool of three different gRNA plasmids with the following sequences: sc-419206 A: Sense: 5′- TAGATAAACTCACCGTGCTA-3′; sc-419206 B: Sense: 5′-CTGCACGTACCCTGAGAATT-3′; sc-419206 C: Sense: 5′-TTACTAGGTACCTTCCATGA-3′. The *Per2-luc* 661W cells cultured on a 12-well plate were co-transfected with 0.5 μg of the BMAL1 CRISPR/Cas9 KO plasmid and 0.5 μg of the BMAL1-R HDR plasmid (Santa Cruz sc-419206-HDR). The plasmid included a puromycin resistance gene (used for the selection of colonies) and the red fluorescent protein (RFP) to detect the correct insertion of the plasmid in the genome. After selection in a medium containing puromycin (4 μg/mL), only cells with RFP signals were isolated. Then the removal of *Bmal1* from the 661W cells was verified by western blotting using the BMAL1 antibody (Cell Signaling Technology, Danvers, MA, USA; cat. no. 14020; RRID:AB_2728705).

### Rescuing *Bmal1* in the 661W- *Bmal1*-KO cells

The plasmid pMSCV-Zeo was purchased from Addgene (Watertown, MA, USA; RRID:Addgene_75088). A 1.1-kb EcoRI to EcoRV fragment containing the expression cassette of the bleomycin-resistant gene driven by the PGK promoter was cut from the pMSCV-Zeo and cloned into the corresponding sites of pCMVSport6 to generate pSport6Zeocin. To re-express *Bmal1* in the 661W- *Bmal1*-KO cells, the *Bmal1* KO cell line was transfected by lipofectamine 2000 (Invitrogen, Waltham, MA, USA) with 250 ng of pCMVFlag *Bmal*1 ^42^ and 25 ng of pSport6Zeocin. Zeocin-resistant colonies were isolated and developed into cell lines. *Bmal1* expression was verified by western blotting.

### Real-time polymerase chain reaction

Cultured 661W cells were collected at different time points and the total RNA was isolated using TRIZOL (Life Technologies). cDNA was synthesized from isolated RNA using a High-Capacity RNA-to-cDNA Kit (Life Technologies). The quantitative polymerase chain reaction (Q-PCR) was performed with the CFX96 Touch Real-Time PCR Detection System (Bio-Rad Laboratories, Hercules, CA, USA) using iQ SYBR Green Supermix (Bio-Rad Laboratories). The primer sequences used to evaluate the gene expression were *Per1* forward, 5′-TGAAGCAAGACCGGGAGA-3′ reverse, 5′-CACACACGCCGTCACATCAA -3′; *Per2* forward, 5′-GAAAGCTGTCACCACCATAGAA-3′ reverse 5′-AACTCGCACTTCCTTTTCAGG-3′; *Bmal1* forward, 5′-AACCTTCCCGCAGCTAACAG-3′ reverse 5′-AGTCCTCTTTGGGCCACCTT-3′; *DBP* forward 5′-CCTGAGGAACAGAAGGATGA-3′ reverse 5′-ATCTGGTTCTCCTTGAGTCTTCTTG-3′; *Nrf2* forward, 5′-CGAGATATACGCAGGAGAGGTAAGA-3′ reverse 5′-GCTCGACAATGTTCTCCAGCTT-3′; 18S ribosomal RNA forward 5′-TTGTTGGTTTTCGGAACTGAGGC-3′ reverse 5′-GGCAAATGCTTTCGCTCTGGTC -3′.

### Drug preparation

Hydrogen peroxide (H _2_O _2_, Sigma-Aldrich, St. Louis, MO, USA) was diluted with double-distilled water to 1 M as a working solution, and 2 μl were added to 2 mL of the medium (final concentration of 1 mM) for two hours. Liproxstatin-1 (Lip1; Cayman Chemical, Ann Arbor, MI, USA) was dissolved in DMSO (47 mM) and further diluted with PBS to 200 μM. Ten microliters of this solution was added to 2 mL of the medium to make 2 μM of the final concentration.

### Measurement of antioxidant capacity and glutathione peroxidase activity

The antioxidant capacity and glutathione peroxidase (GPx) activity were determined using commercially available assay kits (Antioxidant Assay Kit, Cayman Chemical cat. no. 709001; Glutathione Peroxidase Assay Kit, Cayman Chemical cat. no. 703102). The 661W and BKO cells were seeded and grown in 100-mm dishes with 5 mL of medium. When the cells reached 80% to 90% confluency, the medium was exchanged. Cells were washed with PBS and harvested using a cell scraper (CELLTREAT Scientific Products, Pepperell, MA, USA) at 26.5 and 38.5 hours after the medium exchange. Cells were then collected in a 1.5-mL tube, and the numbers of cells in each tube were counted using a cell counter (Bio-Rad TC20). Cells in the tubes were centrifuged (1000 × g for 10 min), and the cell pellet was homogenized in the extraction buffer included in the kits. After centrifuging, the supernatant was collected and the antioxidant capacity or GPx activity was measured according to the manufacturer’s protocol. The microplate reader (Cytation 3; BioTek Instruments, Inc., Winooski, VT, USA) was used to measure the absorbance at 750 and 405 nm for the antioxidant assay and at 340 nm for the GPx assay.

### Western blotting

Cells were grown to near confluence in 35-mm dishes or 6-well plates (Falcon, Fisher Scientific, Waltham, MA, USA), washed with PBS, treated with trypsin, and transferred to 15-mL conical centrifuge tubes (Falcon) with the addition of 5 mL of medium. After centrifugation (400 x g for 5 min), the cell pellets were resuspended in 5 mL of PBS. Cell pellets obtained after another centrifugation (400 x g for 5 min) were lysed with RIPA buffer (Boston BioProducts, Inc., Milford, MA, USA) and supplemented with a protease inhibitor cocktail (cat. no. 5871S, Cell Signaling Technology). Cell lysates were cleared by centrifugation (17,000 x g for 30 min), and aliquots were mixed with Laemmli buffer, 6X SDS-Sample buffer, (cat. no. BP-11R, Boston Bioproducts, Inc.), heated to 95°C, loaded, and run on a Criterion Midi Protein Gel with the Criterion Vertical Electrophoresis Cell System (Bio-Rad Laboratories). Proteins were transferred from gels onto an Amersham HyBond PVDF membrane (Cytiva Life Sciences, Marlborough, MA, USA) using the Bio-Rad Trans-Blot Turbo Transfer system. Membranes were washed with 1X Tris buffered saline (TBS), diluted from a 20X TBS stock solution (Boston Bioproducts, Inc.), for 5 minutes followed by blocking in 5% non-fat dry milk or bovine serum albumin (BSA) (Boston Bioproducts, Inc.) in TBS. The primary antibody in TBST (0.1% Tween-20 in 1X TBS) was then added to the membrane and placed on a rocking platform at 4°C overnight. The membrane was washed in TBST three times for 10 minutes each time on a rotating platform at room temperature before the secondary antibody in TBST was added. Fluorescence-conjugated antibodies were purchased from Fisher Scientific; they included Invitrogen’s goat-anti-rabbit Alexa Fluor Plus 800 (Thermo Fisher Scientific, Inc.; RRID:AB_2633284) or goat-anti-rabbit Alexa Fluor Plus 680 (Thermo Fisher Scientific, Inc.; RRID:AB_2633283), depending on the experiment. After an 1-hour incubation at room temperature, the membrane was washed in TBST three times for 10 minutes each time, after which imaging was done with the LI-COR Odyssey Fc Imaging system (LI-COR, Lincoln, NB, USA).

### Immunohistochemical studies

661W cells were seeded and cultured in chamber slides (Lab-Tek, Inc.) for immunohistochemical studies. Cells were fixed in paraformaldehyde (PAF) 4% and washed with PBS. Cells were incubated for 45 min in 1% BSA and 0.4% Triton-X100 in PBS to permeabilize the membranes and block unspecific binding. Cells were then incubated overnight at 4°C with primary BMAL1 antibodies (1:1500; cat. no. 14020, Cell Signaling Technology), washed in PBS, and incubated for 2 hours at room temperature in secondary antibodies (anti-rabbit conjugated with Alexa Fluor 488, 1:1000). After washing in PBS, slides were cover-slipped with Vectashield (Vector Laboratories, Newark, CA, USA) and cells were visualized with a fluorescence microscope (Zeiss LSM700).

### Statistical analysis

Data were analyzed with a one-way or two-way ANOVA and the Tukey multiple comparison test using SPSS software (IBM; RRID:SCR_016479). A simple paired *t*-test was performed with the Microsoft Excel (RRID:SCR_016137) data analysis program. The rhythmicity of the oxidative stress response was analyzed by the Nitecap program ( https://nitecap.org/: developed by the University of Pennsylvania Perelman School of Medicine). ^43^

## Results

### A functional circadian clock is present in 661W cells.

**Figure 1.**
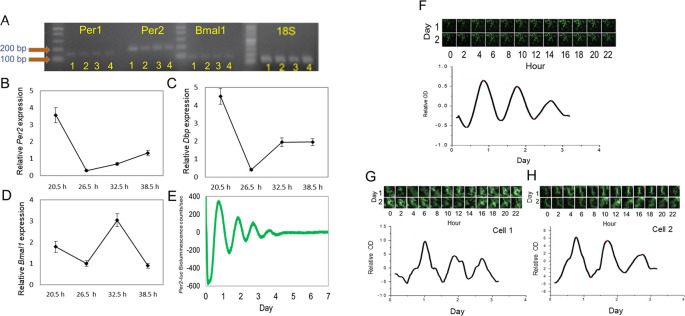
Expression of circadian clock in 661W cells. (A) *Per1, Per2,* and *Bmal1* mRNA was amplified in mouse brain (1), retina (2), RPE (3), and 661W cells (4). (B to D) Circadian expression of *Per2, Bmal1,* and *DBP* mRNA in 661W cells at four different time points (one-way ANOVA, *p* < 0.05 in all cases, n = 6). (E) Representative *Per2-luc* bioluminescence circadian rhythm in 661W cells (detrended data) measured by Lumicycle. (F) *Per2-luc* bioluminescence from 661W cells measured with a CCD camera for 2 days; the bioluminescence rhythm was analyzed. (G to H) A single cell’s *Per2-luc* bioluminescence was measured with a CCD camera.

Cultured 661W cells were grown in a culture dish and collected when they were confluent. RNA was isolated from these cells; *Per1, Per2, Bmal1,* and 18S mRNAs were amplified; and analyzed by electrophoresis. The expression of these genes in the mouse brain, retina, and retinal pigment epithelium (RPE) was also analyzed by electrophoresis to serve as reference points. The results showed that clock genes were expressed in the 661W cells, retina, RPE, and brain ( Figure 1A). To examine the circadian oscillation in the 661W cells, the cells were collected 20.5, 26.5, 32.5, and 38.5 hours after the cells were resynchronized by a medium exchange, and the expression levels of *Per2, Bmal1,* and *DBP* mRNAs were measured. The *Per2* and *DBP* mRNAs were expressed in a circadian manner ( Figure 1B and C, one-way ANOVA, *p* < 0.05), with peak expression at 20.5 hours. *Bmal1* also showed circadian expression ( Figure 1D, one-way ANOVA, *p* < 0.05) with peak expression at 32.5 hours. The bioluminescence emitted from the 661W cells transfected with the *Per2-luc* reporter also showed a circadian rhythm ( Figure 1E). The circadian peak of *Per2-luc* bioluminescence in the 661W cells occurred 20.47 ± 0.21 hours after the medium exchange, and the average circadian period was 23.18 ± 0.15 hours (mean ± SEM, n = 100). The bioluminescence intensity captured by the CCD camera for three days also showed a circadian rhythm ( Figure 1F, peak phase 20.23 hours, period 22.02 hours). A single cell was selected in the area of the CCD image by ImageJ software (RRID:SCR_003070), and a robust *Per2-luc* circadian rhythm was also observed for a single 661W cell ( Figure 1G and H: cell 1 peak: 24.36 hours, period: 20.08 hours; cell 2 peak: 18.43 hours, period 23.90 hours).

### Removal of *Bmal1* abolishes circadian rhythms in 661W cells.

**Figure 2.**
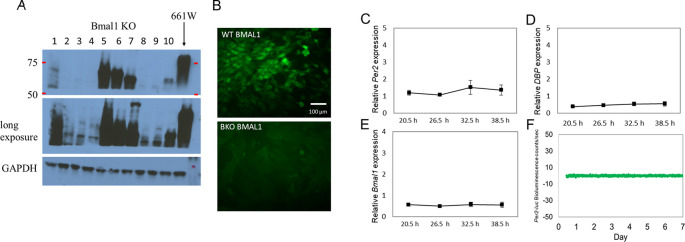
Removal of *Bmal1* from 661W cells abolished circadian oscillation in 661W cells. (A) Western blot indicating the successful knockout of BMAL1 in multiple cell lines (i.e., lines 2, 3, 4, 8, and 9); others show a truncated *Bmal1* (i.e., lines 5, 6, 7, and 10). (B) BMAL1 immunoreactivity was detected in 661W cells (upper panel), but not in a BKO cell line (line 4) (lower panel). (C to D) Loss of rhythmicity in *Per2, DBP,* and *Bmal1* mRNA levels in BKO cells (one-way ANOVA, *p* > 0.05 in all cases, n = 6). (F) 661W-BKO cells no longer exhibit *Per2-luc* bioluminescence rhythm (detrended data) as observed in Figure 1E.

Several *Bmal1* KO (BKO) 661W cell lines were generated using the CRISPR-cas9 gene-editing tool. Western blot analysis showed the successful removal of *Bmal1* in cell lines 2, 3, 4, 8 and 9 ( Figure 2A). A long-exposure image also confirmed the loss of BMAL1 expression. We selected the cell line with the least signal to be used in our experiments. The deletion of *Bmal1* in this line was verified by immunohistochemical studies ( Figure 2B). Consistent with these results, we did not observe any rhythmicity in the expression levels of *Per2, Bmal1,* or *DBP* mRNA in the BKO cells (one-way ANOVA, *p* > 0.1 in all cases; Figure 2C to E) or in the *Per2-luc* bioluminescence ( Figure 2F).

### The circadian clock modulates the sensitivity of 661W cells to oxidative stress.

**Figure 3.**
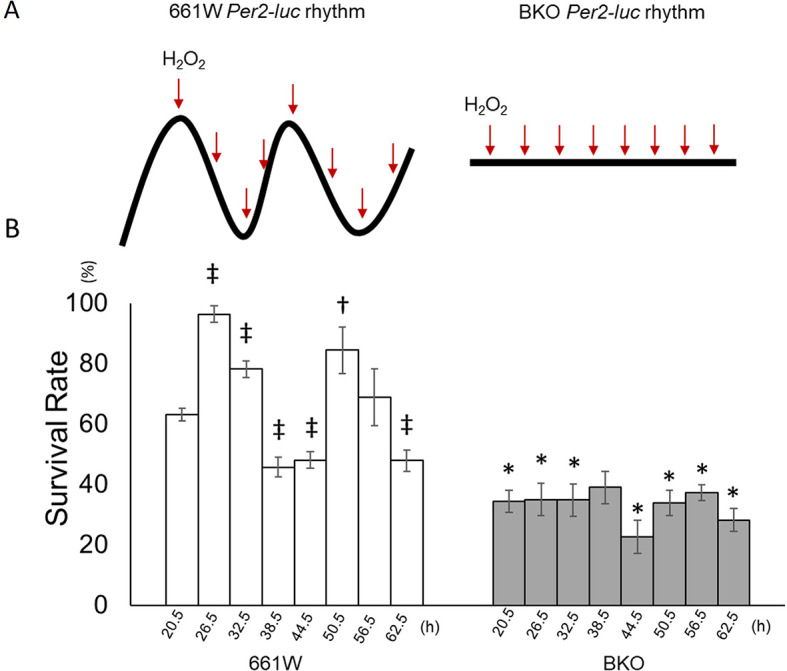
661W cells showed a circadian variation in the response to oxidative stress. (A) Schematic illustration of experimental procedures. Cells were treated with H _2_O _2_ at 6-h intervals from the peak phase of the *Per2-luc* rhythm for two circadian cycles. BKO cells were treated with H _2_O _2_ at the same time points as in 661W cells. (B) The survival rate following exposure to H _2_O _2_ (1 mM) at different circadian times (from 20.5 to 60.5 h) showed a clear circadian rhythm. The survival rate was highest at 26.5 h and lowest at 38.5 h during the first cycle after medium exchange and at 50.6 h and 60.5 h, respectively, during the second cycle. BKO cells did not show circadian variation in oxidative stress sensitivity and had a lower survival rate compared with 661W cells. (Mean ± SEM, n = 7 to 9, two-way ANOVA followed by Tukey multiple comparison test, † *p* < 0.05, ‡ *p* < 0.05 compared with 661W cells at 20.5 h, * *p* < 0.05 compared with same time point for 661W cells).

The 661W and BKO cells were cultured and then subjected to an oxidative stress challenge by adding 1 mM of H _2_O _2_ to the cultured cells for two hours at six-hour intervals starting from the first peak phase of the *Per2-luc* ( Figure 3A) for two consecutive circadian cycles (from 20.5 to 62.5 hours after medium exchange). Cells were harvested two hours after the treatment and then counted. For the control groups, cells were treated with the same volume of double distilled water (n = 6 to 8 for each time point) and the survival rate was normalized with the control value. The 661W cells showed a clear circadian rhythm in their responses to the oxidative stress challenge ( Figure 3B, one-way ANOVA, *p* < 0.05, Jonckheere-Terpstra-Kendall (JTK) algorithm *p* < 0.001: period 24.0 hours: amplitude 0.233, n = 7 to 8). The cells were most resistant to the oxidative stress challenge at 26.5 and 50.5 hours (second cycle) and least resistant at 38.5 and 62.5 hours (second cycle). In the BKO cells, the survival rate was greatly reduced (only ~30% survived the H _2_O _2_ treatment, two-way ANOVA, *p* < 0.01 between groups) and no circadian variation was observed (one-way ANOVA, *p* > 0.05; JTK algorithm *p* > 0.05, Figure 3B).

### *Bmal1* removal affects antioxidant capacity and GPx activity in 661W cells

**Figure 4.**
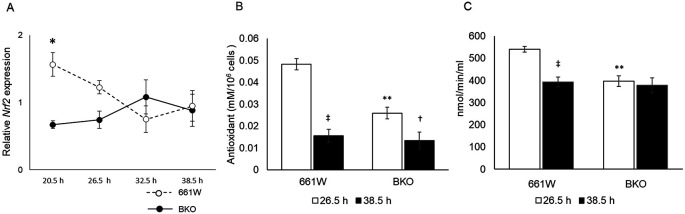
Circadian variation of *Nrf2* mRNA expression and time dependent variation of antioxidant capacity and GPx activity in 661W cells. (A) Circadian expression of *Nrf2* was rhythmic in 661W cells but not in BKO cells (two-way ANOVA, *p* < 0.05 between groups on the Tukey multiple comparison test, *p < 0.05, one-way ANOVA 661W cells: *p* < 0.05; BKO cells: *p* > 0.05, n = 6). (B) Time dependent variation in antioxidant capacity was observed in both WT and BKO cells, although antioxidant capacity of BKO cells was lower than of 661W cells at 26.5 h (two-way ANOVA followed by Tukey multiple comparison test, n = 6, ** *p* < 0.01 661W cells vs. BKO cells, † *p* < 0.05, ‡ *p* < 0.01 26.5 h vs. 38.5 h). (C) Time dependent variation of GPx activity was observed in WT 661W cells but not in BKO cells (two-way ANOVA followed by Tukey multiple comparison test, n = 5 to 6, ** *p* < 0.01 WT cells vs. BKO cells, ‡ *p* < 0.01 26.5 h vs. 38.5 h).

The nuclear factor erythroid 2–related factor 2 ( *Nrf2*) is a well-known regulator for antioxidant response elements, ^44^ and the expression of *Nrf2* is regulated by the circadian clock via E-box elements. ^45^^–^^47^ Hence, we hypothesized that the circadian variation in the sensitivity to oxidative stress was mediated by the circadian regulation of *Nrf2.* Indeed, *Nrf2* mRNA showed a circadian expression in 661W cells ( Figure 4A, one-way ANOVA, *p* < 0.05), whereas in the BKO cells, the expression level of *Nrf2* mRNA was arrhythmic and significantly decreased. We measured the antioxidant capacity in 661W and 661W-BKO cells at the time of maximum and minimum cell survival to the oxidative stress challenge ( *i.e.*, 26.5 and 38.5 hours). In the 661W cells, the antioxidant capacity at 26.5 hours was fourfold higher than the antioxidant capacity at 38.5 hours (two-way ANOVA followed by the Tukey multiple comparison test, *p* < 0.05; Figure 4B). The antioxidant capacity of the 661W-BKO cells was significantly reduced (~50%) with respect to what we observed in the 661W cells at 26.5 hours ( *p* < 0.05). We also observed a slight difference in the antioxidant capacity in the BKO cells between 26.5 and 38.5 hours ( *p* < 0.05; Figure 4B). Of the many antioxidants present in the cells, GPx was a possible candidate for the mediation of the circadian response to oxidative stress because GPx is an intracellular antioxidant that enzymatically reduces H _2_O _2_ to H _2_O. ^48^ Thus, we decided to investigate whether GPx activity was also regulated by the circadian clock in the 661W cells. As shown in Figure 4C, the GPx activity was higher at 26.5 hours and lower at 38.5 hours in the 661W cells (two-way ANOVA followed by Tukey multiple comparison test, *p* < 0.05; Figure 4C). In the BKO cells, the GPx activity was lower at 26.5 hours compared with in the 661W cells (two-way ANOVA followed by Tukey multiple comparison test, *p* < 0.05), and no difference was observed between the level of GPx activity at 26.5 and 38.5 hours.

### Ferroptosis mediated cell death in 661W cells after oxidative stress.

**Figure 5.**
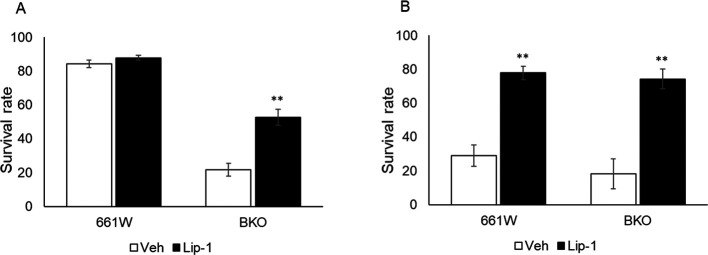
Inhibition of ferroptosis pathway by Lip1 increased cell survival from H _2_O _2_-induced cell death. (A) Treatment of Lip1 increased cell viability by approximately 30% in BKO cells at 26.5 h after medium exchange. (B) Lip1 treatment significantly increased cell viability in both cell types from H _2_O _2_-induced cell death at 38.5 h after medium exchange (two-way ANOVA followed by Tukey multiple comparison test, n = 6, ** *p* < 0.01 control vs. Lip1).

Ferroptosis is a cell death process that is driven by the accumulation of iron-dependent lipid peroxidation. ^49^ GPx plays an important role in this cell death pathway. ^50^ We hypothesized that ferroptosis mediated the cell death process in 661W and BKO cells after H _2_O _2_ exposure. To explore this theory, we investigated whether the ferroptosis inhibitor liproxstatin-1 (Lip1) could prevent cell death from oxidative stress. The 661W and BKO cells were cultured, and pretreated with Lip1 (2 μM) 30 minutes prior to the H _2_O _2_ treatment at 26.5 and 38.5 hours. As shown in Figure 5, treatment with Lip1 increased the survival of BKO cells at 26.5 hours ( Figure 5A) and almost completely prevented cell death at 38.5 hours ( Figure 5B) in both genotypes (two-way ANOVA followed by Tukey multiple comparison test, *p* < 0.01).

### Rescuing the *Bmal1* gene in BKO cells partially restored the circadian rhythm in the sensitivity to oxidative stress

**Figure 6.**
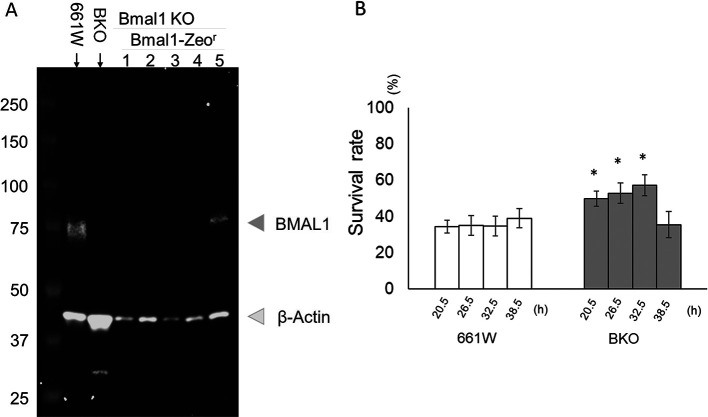
Rescue of *Bmal1* in BKO cells increased cell viability from H _2_O _2_-induced cell death. (A) Western blot indicating the successful re-expression of BMAL1 in the BKO cells (line #5). (B) *Bmal1* rescue restored circadian variation in the sensitivity to oxidative stress in BKO cell (two-way ANOVA followed by Tukey multiple comparison test, n = 6 to 12, * *p* < 0.05 BKO rescued cell vs. BKO cell).

To confirm that the effects we observed in the BKO cells were due to the removal of *Bmal1*, we rescued *Bmal1* in the BKO cells by transfecting the *Bmal1* construct into the BKO cells. The rescued cell lines were confirmed by western blotting ( Figure 6A). Stable BKO- *Bmal1*–rescued cells were cultured and exposed to an oxidative stress challenge as previously described ( Figure 3). Our data indicate that the rescued *Bmal1* in the BKO cells significantly increased the cell survival rate (10% – 20%) during the oxidative stress challenge at 20.5, 26.5, and 32.5 hours (two-way ANOVA followed by Tukey multiple comparison test, * *p* < 0.05; Figure 6B).

## Discussion

The effects of circadian dysfunction caused by genetic and environmental manipulations of the retina have garnered considerable interest in recent years. As previously mentioned, experimental evidence indicated that the cone photoreceptors are very susceptible to circadian disruption. ^4^^,^^5^^,^^12^ However, the lack of a suitable cell model has hampered progress in understanding the molecular mechanism(s) with which the circadian clock may regulate biological functions in these cells. In the present study, we have demonstrated that the cone-like photoreceptor cell line 661W, contains a functional circadian clock; that this circadian clock modulated the response of these cells to an oxidative stress challenge; that the mechanisms by which the circadian clock modulated this response involved the regulation of GPx activity; and that the cell death we observed after H _2_O _2_ exposure was due to ferroptosis. We also observed that removal of the clock gene *Bmal1* from the cell abolished the circadian response and that the rescue of *Bmal1* in the BKO cells increased cell survival after oxidative stress. Thus, we believe that the 661W cell line is a useful new tool for investigating the action of the circadian clock in regulating photoreceptor (cone) biology.

ROS are by-products of metabolic processes and are produced according to the metabolic needs of cells. ^51^ In the retina, the high level of ROS production by photoreceptors occurs during the day when the photoreceptors are actively involved in the phototransduction of light signals. ^52^^,^^53^ Previous studies suggested that circadian dysfunction increases intracellular ROS level ^26^^–^^30^ and that the accumulation of ROS and dysfunction of ROS homeostasis in retinal cells leads to many types of retinal diseases. ^20^ Antioxidants are endogenous products that scavenge excessive ROS. This ROS scavenging system is crucial for cell maintenance and survival. ^22^ Previous studies have shown that the expression patterns of antioxidant enzymes ( *i.e.*, GPx, catalase, peroxiredoxin, and superoxide dismutase) are controlled by the circadian clock. ^54^^–^^58^ Therefore, a functional circadian clock may provide an important protective function by synchronizing the production of the antioxidant response with the time of maximum ROS production, thus reducing the numbers of ROS in a cell. Our new data supported this notion by demonstrating that in 661W cells, the circadian clock can modulate the response to oxidative stress by regulating the antioxidant capacity ( Figures 3 and 4B).

A number of studies have suggested that the involvement of the circadian clock in the regulation of antioxidant elements is mediated through *Nrf2*. ^58^^,^^59^ Indeed, previous investigations have shown that the circadian expression of *Nrf2* is under the direct control of the circadian clock via the E-box present on the promoter region of this gene and that *Nrf2* shows a similar pattern of expression of *Per1* and *Per2*. ^12^^,^^45^ Our results agree with these previous studies, showing that *Nrf2* mRNA is rhythmically transcribed with a similar rhythmic pattern of *Per2* in 661W cells but not in BKO cells.

One of the most surprising results obtained in our study regarding the antioxidant capacity of 661-BKO cells is the presence of a circadian rhythm (although at a much lower amplitude than 661W cells) ( Figure 4A). A previous study showed that peroxiredoxins, a group of antioxidant proteins, may have a circadian rhythm in redox cycles in red blood cells, ^57^ which have no nucleus and therefore cannot generate a circadian oscillation via the transcriptional translational feedback loops’ clock mechanism. ^60^ Additionally, another recent study has reported that circadian oscillation in the transcriptome and proteome of mammals can be present in the fibroblast and in the mouse liver even when *Bmal1* has been removed. ^61^ Although the results reported in these studies are controversial, ^62^^,^^63^ our new data may suggest that in some situations, the removal of *Bmal1* may not completely abolish circadian oscillations ( Figure 4B). However, it is important to notice that although the antioxidant capability has a circadian variation in 661W-BKO cells, the changes did not extend to changes in the GPx antioxidant capability ( Figure 4C) and, more importantly, did not translate to cell survivability after an oxidative stress challenge ( Figure 3B).

Ferroptosis is a newly discovered nonapoptotic cell death cascade that is characteristic of the iron-dependent oxidation of phospholipids. ^48^ Ferroptosis is activated by the dysregulation of the GPx antioxidant defense mechanism, which causes lipid peroxidation and cell death. ^64^ Circadian expression of GPx has been reported in many organs, including the mouse retina, ^45^^,^^58^ and accumulating evidence suggests that the dysregulation of GPx causes retinal diseases and blindness. ^65^^,^^66^ Our results showed that Lip-1 (inhibiting lipid peroxidation to prevent ferroptosis activation ^67^) prevents cell death from oxidative stress at 38.5 hours in both genotypes, thus suggesting that the cell death that follows H _2_O _2_ exposure is probably due to ferroptosis. However, it may be that other cell death pathways ( *i.e.*, apoptosis, necroptosis ^68^^,^^69^) are involved, since Lip-1 only partially prevented cell death in BKO cells at 26.5 hours.

Finally, it is important to mention that *in vivo* studies have shown that the magnitude of oxidative stress damage (light-induced photoreceptor damage) is greatly affected by the time of the day. Nocturnal rodents are three to four times more susceptible to light damage at night than during the day. ^70^^–^^72^ The circadian dependence of light-induced photoreceptor damage appears to involve changes in the cAMP levels. ^73^ Our new data expands these previous results by suggesting that a change in GPx may be involved in the modulation of the circadian dependence of light-induced photoreceptor damage.

## Conclusion

Our work supports the notion that the presence of a functional circadian clock and its ability to modulate the response to an oxidative stress is the undelaying mechanism that may protect cones during aging.

## Author contributions

KB, T-CS, VG, AS, AD and JD performed experiments and analyzed experimental results. KB and GT planned/designed the studies, provided data/figures, and wrote the manuscript.

## Data Availability

Figshare: The circadian clock mediates the response to oxidative stress in a cone photoreceptor–like (661W) cell line via regulation of glutathione peroxidase activity, https://doi.org/10.6084/m9.figshare.20537982.v1. ^74^
• qPCR CT values for circadian gene expressions in 661W cells •
Figure 1 clock gene expressions in 661W cells Figshare: The circadian clock mediates the response to oxidative stress in a cone photoreceptor–like (661W) cell line via regulation of glutathione peroxidase activity, https://doi.org/10.6084/m9.figshare.20538408.v1. ^75^
• The peak phases and circadian periods of Per2-luc biouminescence rhythms in 661W cells • Per2-luc phases and periods in 661W cells Figshare: The circadian clock mediates the response to oxidative stress in a cone photoreceptor–like (661W) cell line via regulation of glutathione peroxidase activity, https://doi.org/10.6084/m9.figshare.20538621.v1. ^76^
• Slide 1: CCD recording from population of 661W cells. Slide 2-4: Single cell recording. Slide 4 may show circadian rhythms of multiple cells since the first peak shows two peaks • Per2-luc bioluminescence from 661W cells measured with a CCD camera Figshare: The circadian clock mediates the response to oxidative stress in a cone photoreceptor–like (661W) cell line via regulation of glutathione peroxidase activity, https://doi.org/10.6084/m9.figshare.20538711.v1. ^77^
• qPCR CT values for circadian gene expressions in Bmal1 KO cells •
Figure 2 Circadian gene expressions in BKO cells Figshare: The circadian clock mediates the response to oxidative stress in a cone photoreceptor–like (661W) cell line via regulation of glutathione peroxidase activity, https://doi.org/10.6084/m9.figshare.20538885.v1. ^78^
• The cell survival rate following exposure to H _2_O _2_ or vehicle control treatment in 661W and BKO cells •
Figure 3 Cell viability from oxidative stress challenge Figshare: The circadian clock mediates the response to oxidative stress in a cone photoreceptor–like (661W) cell line via regulation of glutathione peroxidase activity, https://doi.org/10.6084/m9.figshare.20538975.v1. ^79^
• Circadian variation of cell survival rate was analyzed by Nitecap • JTK analysis by Nitecap Figshare: The circadian clock mediates the response to oxidative stress in a cone photoreceptor–like (661W) cell line via regulation of glutathione peroxidase activity, https://doi.org/10.6084/m9.figshare.20539026.v1. ^80^
• Data sets for Nrf2 circadian expression in 661W and BKO cells and circadia variation for GPx activity level •
Figure 4 A and C data set Nrf2 expression and GPx activity level in 661W and BKO cells Figshare: The circadian clock mediates the response to oxidative stress in a cone photoreceptor–like (661W) cell line via regulation of glutathione peroxidase activity, https://doi.org/10.6084/m9.figshare.20539398.v1. ^81^
• The spectrometry counts obtained from antioxidant assay in 661W and BKO cells •
Figure 4 B data set Measurement of antioxidant capacity in 661W and BKO cells Figshare: The circadian clock mediates the response to oxidative stress in a cone photoreceptor–like (661W) cell line via regulation of glutathione peroxidase activity, https://doi.org/10.6084/m9.figshare.20539485.v1. ^82^
• The survival rate after Lip1 or vehicle treatment in 661W and BKO cells at two time points •
Figure 5 The survival rate after pretreatment of Lip1 followed by oxidative stress challenge Figshare: The circadian clock mediates the response to oxidative stress in a cone photoreceptor–like (661W) cell line via regulation of glutathione peroxidase activity, https://doi.org/10.6084/m9.figshare.20539737.v1. ^83^
• The data set for the survival rate in Bmal1 rescued cells following oxidative stress challenge •
Figure 6 The cell viability in Bmal1 rescued cells following oxidative stress challenge Data are available under the terms of the Creative Commons Zero “No rights reserved” data waiver (CC0 1.0 Public domain dedication).

## References

[1] Tosini G Menaker M : Circadian rhythms in cultured mammalian retina. *Science.* 1996;272:419–421. 10.1126/science.272.5260.419 8602533

[2] Tosini G Menaker M : The clock in the mouse retina: melatonin synthesis and photoreceptor degeneration. *Brain Res.* 1998;789:221–228. 10.1016/S0006-8993(97)01446-7 9573370

[3] Felder-Schmittbuhl MP Buhr ED Dkhissi-Benyahya O : Ocular clocks: Adapting mechanisms for eye functions and health. *Vis. Sci.* 2018;59(12):4856–4870. 10.1167/iovs.18-24957 PMC6181243 30347082

[4] Storch KF Paz C Signorovitch J : Intrinsic circadian clock of the mammalian retina: importance for retinal processing of visual information. *Cell.* 2007;130:730–741. 10.1016/j.cell.2007.06.045 17719549 PMC2040024

[5] Baba K Ribelayga CP Michael Iuvone P : The Retinal Circadian Clock and Photoreceptor Viability. *Adv. Exp. Med. Biol.* 2018;1074:345–350. 10.1007/978-3-319-75402-4_42 29721962 PMC6003627

[6] Ait-Hmyed O Felder-Schmittbuhl MP : Mice lacking Period 1 and Period 2 circadian clock genes exhibit blue cone photoreceptor defects. *Eur. J. Neurosci.* 2013;37:1048–1060. 10.1111/ejn.12103 23351077

[7] DeVera C Dixon J Chrenek MA : The Circadian Clock in the Retinal Pigment Epithelium Controls the Diurnal Rhythm of Phagocytic Activity. *Int. J. Mol. Sci.* 2022;23(10):5302. 10.3390/ijms23105302 35628111 PMC9141420

[8] Ruan GX Allen GC Yamazaki S : An Autonomous Circadian Clock in the Inner Mouse Retina Regulated by Dopamine and GABA. *PLoS Biol.* 2008;6(10):1–18. 10.1371/journal.pbio.0060249 18959477 PMC2567003

[9] Liu X Zhang Z Ribelayga CP : Heterogeneous expression of the core circadian clock proteins among neuronal cell types in mouse retina. *PLoS One.* 2012;7:e50602. 10.1371/journal.pone.0050602 23189207 PMC3506613

[10] Hiragaki S Baba K Coulson E : Melatonin Signaling Modulates Clock Genes Expression in the Mouse Retina. *PLoS One.* 2014;9(9):e106819. 10.1371/journal.pone.0106819 25203735 PMC4159264

[11] Jaeger C Sandu C Malan A : Circadian organization of the rodent retina involves strongly coupled, layer-specific oscillators. *FASEB J.* 2015;29(4):1493–1504. 10.1096/fj.14-261214 25573753

[12] Sawant OB Horton AM Zucaro OF : The Circadian Clock Gene Bmal1 Controls Thyroid Hormone-Mediated Spectral Identity and Cone Photoreceptor Function. *Cell Rep.* 2017;21(3):692–706. 10.1016/j.celrep.2017.09.069 29045837 PMC5647869

[13] Baba K Piano I Lyuboslavsky P : Removal of clock gene Bmal1 from the retina affects retinal development and accelerates cone photoreceptor degeneration during aging. *Proc. Natl. Acad. Sci. U. S. A.* 2018;115:13099–13104. 10.1073/pnas.1808137115 30498030 PMC6305005

[14] DeVera C Tosini G : Circadian analysis of the mouse retinal pigment epithelium transcriptome. *Exp. Eye Res.* 2020;193:107988. 10.1016/j.exer.2020.107988 32105725 PMC7372111

[15] Ames A 3rd Li YY Heher EC : Energy metabolism of rabbit retina as related to function: high cost of Na+ transport. *J. Neurosci.* 1992;12:840–853. 10.1523/JNEUROSCI.12-03-00840.1992 1312136 PMC6576058

[16] Punzo C Xiong W Cepko CL : Loss of daylight vision in retinal degeneration: are oxidative stress and metabolic dysregulation to blame? *J. Biol. Chem.* 2012;287:1642–1648. 10.1074/jbc.R111.304428 22074929 PMC3265845

[17] Hoang QV Linsenmeier RA Chung CK : Photoreceptor inner segments in monkey and human retina: mitochondrial density, optics, and regional variation. *Vis. Neurosci.* 2002;19:395–407. 10.1017/S0952523802194028 12511073

[18] Perkins GA Ellisman MH Fox DA : Three-dimensional analysis of mouse rod and cone mitochondrial cristae architecture: bioenergetic and functional implications. *Mol. Vis.* 2003;9:60–73. 12632036

[19] Campos PB Paulsen BS Rehen SK : Accelerating neuronal aging in in vitro model brain disorders: a focus on reactive oxygen species. *Front. Aging Neurosci.* 2014;6:292. 25386139 10.3389/fnagi.2014.00292 PMC4209886

[20] Eells JT : Mitochondrial Dysfunction in the Aging Retina. *Biology (Basel).* 2019;8(2):31. 10.3390/biology8020031 31083549 PMC6627398

[21] Guo C Sun L Chen X : Oxidative stress, mitochondrial damage and neurodegenerative diseases. *Neural Regen. Res.* 2013;8(21):2003–2014. 10.3969/j.issn.1673-5374.2013.21.009 25206509 PMC4145906

[22] Schieber M Chandel NS : ROS Function in Redox Signaling and Oxidative Stress. *Curr. Biol.* 2014;24(10):R453–R462. 10.1016/j.cub.2014.03.034 24845678 PMC4055301

[23] Diaz-Munoz M Hernandez-Munoz R Suarez J : Day-night cycle of lipid peroxidation in rat cerebral cortex and their relationship to the glutathione cycle and superoxide dismutase activity. *Neuroscience.* 1985;16:859–863. 10.1016/0306-4522(85)90100-9 4094696

[24] Hardeland R Coto-Montes A Poeggeler B : Circadian rhythms, oxidative stress, and antioxidative defense mechanisms. *Chronobiol. Int.* 2003;20:921–962. 10.1081/CBI-120025245 14680136

[25] Kondratov RV Kondratova AA Gorbacheva VY : Early aging and age-related pathologies in mice deficient in BMAL1, the core component of the circadian clock. *Genes Dev.* 2006;20:1868–1873. 10.1101/gad.1432206 16847346 PMC1522083

[26] Kondratov RV Vykhovanets O Kondratova AA : Antioxidant N-acetyl-L-cysteine ameliorates symptoms of premature aging associated with the deficiency of the circadian protein BMAL1. *Aging.* 2009;1:979–987. 10.18632/aging.100113 20157581 PMC2815755

[27] Anea CB Cheng B Sharma S : Increased superoxide and endothelial NO synthase uncoupling in blood vessels of Bmal1-knockout mice. *Circ. Res.* 2012;111:1157–1165. 10.1161/CIRCRESAHA.111.261750 22912383 PMC3740771

[28] Anea CB Zhang M Chen F : Circadian clock control of Nox4 and reactive oxygen species in the vasculature. *PLoS One.* 2013;8:e78626. 10.1371/journal.pone.0078626 24205282 PMC3808297

[29] Musiek ES Lim MM Yang G : Circadian clock proteins regulate neuronal redox homeostasis and neurodegeneration. *J. Clin. Invest.* 2013;123:5389–5400. 10.1172/JCI70317 24270424 PMC3859381

[30] Jacobi D Liu S Burkewitz K : Hepatic Bmal1 Regulates Rhythmic Mitochondrial Dynamics and Promotes Metabolic Fitness. *Cell Metab.* 2015;22:709–720. 10.1016/j.cmet.2015.08.006 26365180 PMC4598294

[31] Tan E Ding XQ Saadi A : Expression of cone-photoreceptor-specific antigens in a cell line derived from retinal tumors in transgenic mice. *Invest. Ophthalmol. Vis. Sci.* 2004;45:764–768. 10.1167/iovs.03-1114 14985288 PMC2937568

[32] Al-Ubaidi MR Matsumoto H Kurono S : Proteomics profiling of the cone photoreceptor cell line, 661W. *Adv. Exp. Med. Biol.* 2008;613:301–311. 10.1007/978-0-387-74904-4_35 18188958

[33] Layton CJ : Diabetic levels of glucose increase cellular reducing equivalents but reduce survival in three models of 661W photoreceptor-like cell injury. *BMC Ophthalmol.* 2015;15:174. 10.1186/s12886-015-0164-2 26653778 PMC4675021

[34] Natoli R Rutar M Lu YZ : The Role of Pyruvate in Protecting 661W Photoreceptor-Like Cells Against Light-Induced Cell Death. *Curr. Eye Res.* 2016;41(11):1473–1481. 10.3109/02713683.2016.1139725 27217092

[35] Lin JB Kubota S Ban N : NAMPT-Mediated NAD(+) Biosynthesis Is Essential for Vision in Mice. *Cell Rep.* 2016;17:69–85. 10.1016/j.celrep.2016.08.073 27681422 PMC5104206

[36] Chen WJ Wu C Xu Z : Nrf2 protects photoreceptor cells from photo-oxidative stress induced by blue light. *Exp. Eye Res.* 2017;154:151–158. 10.1016/j.exer.2016.12.001 27923559 PMC6054877

[37] Sánchez-Bretaño A Baba K Janjua U : Melatonin partially protects 661W cells from H2O2-induced death by inhibiting Fas/FasL-caspase-3. *Mol. Vis.* 2017;23:844–852. 29259391 PMC5723148

[38] DeBruyne JP Baggs JE Sato TK : Ubiquitin ligase Siah2 regulates RevErbα degradation and the mammalian circadian clock. *Proc. Natl. Acad. Sci. U. S. A.* 2015;112(40):12420–12425. 10.1073/pnas.1501204112 26392558 PMC4603519

[39] Baba K Davidson AJ Tosini G : Melatonin Entrains PER2::LUC Bioluminescence Circadian Rhythm in the Mouse Cornea. *Invest. Ophthalmol. Vis. Sci.* 2015;56:4753–4758. 10.1167/iovs.15-17124 26207312 PMC4516012

[40] Evans JA Davidson AJ : Health consequences of circadian disruption in humans and animal models. *Prog. Mol. Biol. Transl. Sci.* 2013;119:283–323. 10.1016/B978-0-12-396971-2.00010-5 23899601

[41] Baba K Sengupta A Tosini M : Circadian regulation of the PERIOD 2::LUCIFERASE bioluminescence rhythm in the mouse retinal pigment epithelium-choroid. *Mol. Vis.* 2010;16:2605–2611. 21151601 PMC3000237

[42] Sato TK Yamada RG Ukai H : Feedback repression is required for mammalian circadian clock function. *Nat. Genet.* 2006;38(3):312–319. 10.1038/ng1745 16474406 PMC1994933

[43] Brooks TG Mrčela A Lahens NF : Nitecap: An Exploratory Circadian Analysis Web Application. *J. Biol. Rhythm.* 2022;37(1):43–52. 10.1177/07487304211054408 34724846 PMC9003665

[44] Nguyen T Nioi P Pickett CB : The Nrf2-antioxidant response element signaling pathway and its activation by oxidative stress. *J. Biol. Chem.* 2009;284(20):13291–13295. 10.1074/jbc.R900010200 19182219 PMC2679427

[45] Pekovic-Vaughan V Gibbs J Yoshitane H : The circadian clock regulates rhythmic activation of the NRF2/glutathione-mediated antioxidant defense pathway to modulate pulmonary fibrosis. *Genes Dev.* 2014;28(6):548–560. 10.1101/gad.237081.113 24637114 PMC3967045

[46] Early JO Menon D Wyse CA : Circadian clock protein BMAL1 regulates IL-1β in macrophages via NRF2. *Proc. Natl. Acad. Sci. U. S. A.* 2018;115(36):E8460–E8468. 10.1073/pnas.1800431115 30127006 PMC6130388

[47] Wible RS Ramanathan C Sutter CH : NRF2 regulates core and stabilizing circadian clock loops, coupling redox and timekeeping in Mus musculus. *elife.* 2018;7:e31656. 10.7554/eLife.31656 29481323 PMC5826263

[48] Ng CF Schafer FQ Buettner GR : The rate of cellular hydrogen peroxide removal shows dependency on GSH: Mathematical insight into in vivo H _2_O _2_ and GPx concentrations. *Free Radic. Res.* 2007;41(11):1201–1211. 10.1080/10715760701625075 17886026 PMC2268624

[49] Dixon SJ Lemberg KM Lamprecht MR : Ferroptosis: an iron-dependent form of nonapoptotic cell death. *Cell.* 2012;149(5):1060–1072. 10.1016/j.cell.2012.03.042 22632970 PMC3367386

[50] Yang WS Stockwell BR : Ferroptosis: Death by Lipid Peroxidation. *Trends Cell Biol.* 2016;26(3):165–176. 10.1016/j.tcb.2015.10.014 26653790 PMC4764384

[51] Sinenko SA Starkova TY Kuzmin AA : Physiological Signaling Functions of Reactive Oxygen Species in Stem Cells: From Flies to Man. *Front. Cell Dev. Biol.* 2021;9:714370. 10.3389/fcell.2021.714370 34422833 PMC8377544

[52] Chang JY Shi L Ko ML : Circadian Regulation of Mitochondrial Dynamics in Retinal Photoreceptors. *J. Biol. Rhythm.* 2018;33(2):151–165. 10.1177/0748730418762152 29671706

[53] Giarmarco MM Brock DC Robbings BM : Daily mitochondrial dynamics in cone photoreceptors. *Proc. Natl. Acad. Sci. U. S. A.* 2020;117(46):28816–28827. 10.1073/pnas.2007827117 33144507 PMC7682359

[54] Reddy AB Karp NA Maywood ES : Circadian orchestration of the hepatic proteome. *Curr. Biol.* 2006;16:1107–1115. 10.1016/j.cub.2006.04.026 16753565

[55] Fonzo LS Golini RS Delgado SM : Temporal patterns of lipoperoxidation and antioxidant enzymes are modified in the hippocampus of vitamin A-deficient rats. *Hippocampus.* 2009;19:869–880. 10.1002/hipo.20571 19308957 PMC3217053

[56] Sani M Sebaï H Gadacha W : Catalase activity and rhythmic patterns in mouse brain, kidney and liver. *Comp. Biochem. Physiol. B Biochem. Mol. Biol.* 2006;145:331–337. 10.1016/j.cbpb.2006.08.005 17045502

[57] O’Neill JS Reddy AB : Circadian clocks in human red blood cells. *Nature.* 2011;469:498–503. 10.1038/nature09702 21270888 PMC3040566

[58] Xu YQ Zhang D Jin T : Diurnal variation of hepatic antioxidant gene expression in mice. *PLoS One.* 2012;7:e44237. 10.1371/journal.pone.0044237 22952936 PMC3430632

[59] Patel SA Velingkaar NS Kondratov RV : Transcriptional control of antioxidant defense by the circadian clock. *Antioxid. Redox Signal.* 2014;20(18):2997–3006. 10.1089/ars.2013.5671 24111970 PMC4038985

[60] Takahashi JS : Transcriptional architecture of the mammalian circadian clock. *Nat. Rev. Genet.* 2017;18(3):164–179. 10.1038/nrg.2016.150 27990019 PMC5501165

[61] Ray S Valekunja UK Stangherlin A : Circadian rhythms in the absence of the clock gene Bmal1. *Science.* 2020;367(6479):800–806. 10.1126/science.aaw7365 32054765

[62] Abruzzi KC Gobet C Naef F : Comment on “Circadian rhythms in the absence of the clock gene Bmal1”. *Science.* 2021;372(6539):eabf0922. 10.1126/science.abf0922 33859000

[63] Ness-Cohn E Allada R Braun R : Comment on “Circadian rhythms in the absence of the clock gene Bmal1”. *Science.* 2021;372(6539):eabe9230. 10.1126/science.abe9230 33859007 PMC9172996

[64] Cao JY Dixon SJ : Mechanisms of ferroptosis. *Cell. Mol. Life Sci.* 2016;73(11-12):2195–2209. 10.1007/s00018-016-2194-1 27048822 PMC4887533

[65] Ohira A Tanito M Kaidzu S : Glutathione peroxidase induced in rat retinas to counteract photic injury. *Invest. Ophthalmol. Vis. Sci.* 2003;44(3):1230–1236. 10.1167/iovs.02-0191 12601053

[66] Lu L Oveson BC Jo YJ : Increased expression of glutathione peroxidase 4 strongly protects retina from oxidative damage. *Antioxid. Redox Signal.* 2009;11(4):715–724. 10.1089/ars.2008.2171 18823256 PMC2787833

[67] Zilka O Shah R Li B : On the Mechanism of Cytoprotection by Ferrostatin-1 and Liproxstatin-1 and the Role of Lipid Peroxidation in Ferroptotic Cell Death. *ACS Cent. Sci.* 2017;3(3):232–243. 10.1021/acscentsci.7b00028 28386601 PMC5364454

[68] Hanus J Zhang H Wang Z : Induction of necrotic cell death by oxidative stress in retinal pigment epithelial cells. *Cell Death Dis.* 2013;4(12):e965. 10.1038/cddis.2013.478 24336085 PMC3877549

[69] Redza-Dutordoir M Averill-Bates DA : Activation of apoptosis signalling pathways by reactive oxygen species. *Biochim. Biophys. Acta.* 2016;1863(12):2977–2992. 10.1016/j.bbamcr.2016.09.012 27646922

[70] Duncan TE O’Steen WK : The diurnal susceptibility of rat retinal photoreceptors to light-induced damage. *Exp. Eye Res.* 1985;41(4):497–507. 10.1016/S0014-4835(85)80007-5 4085578

[71] White MP Fisher LJ : Degree of light damage to the retina varies with time of day of bright light exposure. *Physiol. Behav.* 1987;39(5):607–613. 10.1016/0031-9384(87)90160-0 3588706

[72] Organisciak DT Darrow RM Barsalou L : Circadian-dependent retinal light damage in rats. *Invest. Ophthalmol. Vis. Sci.* 2000;41(12):3694–3701. 11053264

[73] Humphries A Carter DA : Circadian dependency of nocturnal immediate-early protein induction in rat retina. *Biochem. Biophys. Res. Commun.* 2004;320(2):551–556. 10.1016/j.bbrc.2004.06.006 15219864

[74] Baba K : Figure 1 clock gene expressions in 661W cells. figshare.Dataset.2022. 10.6084/m9.figshare.20537982.v1

[75] Baba K : Per2-luc phases and periods in 661W cells. figshare.Dataset.2022. 10.6084/m9.figshare.20538408.v1

[76] Baba K : Per2-luc bioluminescence from 661W cells measured with a CCD camera. figshare.Media.2022. 10.6084/m9.figshare.20538621.v1

[77] Baba K : Figure 2 Circadian gene expressions in BKO cells. figshare.Dataset.2022. 10.6084/m9.figshare.20538711.v1

[78] Baba K : Figure 3 Cell viability from oxidative stress challenge. figshare.Dataset.2022. 10.6084/m9.figshare.20538885.v1

[79] Baba K : JTK analysis by Nitecap. figshare.Figure.2022. 10.6084/m9.figshare.20538975.v1

[80] Baba K : Figure 4 A and C data set Nrf2 expression and GPx activity level in 661W and BKO cells. figshare.Dataset.2022. 10.6084/m9.figshare.20539026.v1

[81] Baba K : Figure 4 B data set Measurement of antioxidant capacity in 661W and BKO cells. figshare.Dataset.2022. 10.6084/m9.figshare.20539398.v1

[82] Baba K : Figure 5 The survival rate after pretreatment of Lip1 followed by oxidative stress challenge. figshare.Dataset.2022. 10.6084/m9.figshare.20539485.v1

[83] Baba K : Figure 6 The cell viability in Bmal1 rescued cells following oxidative stress challenge. figshare.Dataset.2022. 10.6084/m9.figshare.20539737.v1

