# Supplementary material for: The circadian clock mediates the response to oxidative stress in a cone photoreceptor‒like (661W) cell line via regulation of glutathione peroxidase activity
Source: F1000Res. 2022 Nov 21;11:1072. Originally published 2022 Sep 20. [Version 2] doi: 10.12688/f1000research.125133.2 (PMC9639596; doi:10.12688/f1000research.125133.2)
Supplement: Peer review history

# Peer review: Reviewer response for version 2

**DOI:** 10.5256/f1000research.141194.r156226

**Published:** 2022-11-23

**Authors:** Ethan D. Buhr

**Affiliations:** 1 Department of Ophthalmology, University of Washington School of Medicine, Seattle, WA, USA

No further comments.

Is the work clearly and accurately presented and does it cite the current literature?

Yes

If applicable, is the statistical analysis and its interpretation appropriate?

Yes

Are all the source data underlying the results available to ensure full reproducibility?

Yes

Is the study design appropriate and is the work technically sound?

Yes

Are the conclusions drawn adequately supported by the results?

Yes

Are sufficient details of methods and analysis provided to allow replication by others?

Yes

Reviewer Expertise:

retina, cornea, opsins, circadian rhythms

I confirm that I have read this submission and believe that I have an appropriate level of expertise to confirm that it is of an acceptable scientific standard.

# Peer review: Reviewer response for version 2

**DOI:** 10.5256/f1000research.141194.r156225

**Published:** 2022-11-21

**Authors:** Sujata Rao

**Affiliations:** 1 Department of Ophthalmic Research, Cole Eye Institute, Cleveland Clinic, Cleveland, OH, USA

No further comments.

Is the work clearly and accurately presented and does it cite the current literature?

Yes

If applicable, is the statistical analysis and its interpretation appropriate?

Yes

Are all the source data underlying the results available to ensure full reproducibility?

Yes

Is the study design appropriate and is the work technically sound?

Yes

Are the conclusions drawn adequately supported by the results?

Yes

Are sufficient details of methods and analysis provided to allow replication by others?

Yes

Reviewer Expertise:

Retina, circadian rhythms, opsins, cone photoreceptor.

I confirm that I have read this submission and believe that I have an appropriate level of expertise to confirm that it is of an acceptable scientific standard.

# Peer review: Reviewer response for version 1

**DOI:** 10.5256/f1000research.137406.r151007

**Published:** 2022-11-03

**Authors:** Sujata Rao

**Affiliations:** 1 Department of Ophthalmic Research, Cole Eye Institute, Cleveland Clinic, Cleveland, OH, USA

This study aims to delineate the molecular mechanisms regulated by the circadian clock in the aging cone photoreceptors. This study is a continuation of a previous finding from this group, demonstrating the role of the circadian clock in cone photoreceptor viability during aging. In the current study, the authors have utilized a mouse-derived cone-like cell, 661W, to assess the effects of oxidative stress on these cells. The authors demonstrate that 661W cells contain a functional circadian clock that mediates the response to oxidative stress in vitro. This antioxidant mechanism is abolished when Bmal1 is deleted from these cells. Furthermore, reintroducing Bmal1 restores the circadian response to oxidative stress, providing supporting evidence that Bmal1 mediates the effects. These findings are intriguing and provide additional evidence for the importance of the circadian clock in regulating cellular responses to stress. The data is robust and will be of interest to the vision researchers.

 There are a few minor points that the authors should clarify

1. In the method section, include how the survival was assessed. Was it the total number of cells or dying cells?
2. Interestingly, the highest level of Bmal1 transcript is observed at 32.5h. Yet both the maximal oxidative stress-induced survival rate and peak Nrf2 expression occurs at 26.5h when Bmal1 expression is the lowest. Can the authors comment on that?
3. Could the authors clarify the duration of the H202 and how long after the exposure the cells were harvested for the various assessments?
4. In the discussion, it wasn’t clear why the authors state that “removal of Bmal1 may not completely abolish circadian oscillations based on Figure4B”. The data provided is not sufficient to make that conclusion.  

Is the work clearly and accurately presented and does it cite the current literature?

Yes

If applicable, is the statistical analysis and its interpretation appropriate?

Yes

Are all the source data underlying the results available to ensure full reproducibility?

Yes

Is the study design appropriate and is the work technically sound?

Yes

Are the conclusions drawn adequately supported by the results?

Yes

Are sufficient details of methods and analysis provided to allow replication by others?

Yes

Reviewer Expertise:

Retina, circadian rhythms, opsins, cone photoreceptor.

I confirm that I have read this submission and believe that I have an appropriate level of expertise to confirm that it is of an acceptable scientific standard.

# Response

**Published:** 2022-11-09

**Authors:** Kenkichi Baba

**Affiliations:** Morehouse School of Medicine, USA

Thank you Dr. Rao for the Valuable comments and suggestions

*Comment: In the method section, include how the survival was assessed. Was it the total number of cells or dying cells?*

 Response: Thank you for mentioning. The cell survival rate was calculated from the number of living cells out of total cell counts.  We added it in the methods.

*Comment: Interestingly, the highest level of Bmal1 transcript is observed at 32.5h. Yet both the maximal oxidative stress-induced survival rate and peak Nrf2 expression occurs at 26.5h when Bmal1 expression is the lowest. Can the authors comment on that?*

 Response: Several studies indicate expression level of *Nrf2* is regulated via e-box in its promoter and shows similar rhythmic trend as *Per1* and *Per2* expressions. We believe that the circadian clock directly drives *Nrf2* expression and that translates to survival rate against oxidative stress.

*Comment: Could the authors clarify the duration of the H _2_0 _2_ and how long after the exposure the cells were harvested for the various assessments?*

 Response: Thank you for mentioning. For oxidative stress challenge experiment, we treated cells with hydrogen peroxide for two hours and cells were harvested after two hours of treatment for cell counts. We have added details in Methods.

*Comment: In the discussion, it wasn’t clear why the authors state that “removal of Bmal1 may not completely abolish circadian oscillations based on Figure4B”. The data provided is not sufficient to make that conclusion.*

 Response: Thank you for your comment. We agree that only two-time points data is not sufficient to conclude it as circadian rhythmicity. We rephrased it in the discussion.

# Peer review: Reviewer response for version 1

**DOI:** 10.5256/f1000research.137406.r151006

**Published:** 2022-10-04

**Authors:** Ethan D. Buhr

**Affiliations:** 1 Department of Ophthalmology, University of Washington School of Medicine, Seattle, WA, USA

Baba *et al.* show that the 661W cell line expresses a functional molecular circadian clock which is lost with the deletion of *Bmal1*. The circadian oscillation of cellular viability in the face of oxidative chemical stress is lost in *Bmal1KO* cells, as well as an overall loss in cellular robustness (as nearly all time points have reduced cell survival compared to WT).

 The authors then show that a rhythm of transcript abundance of the antioxidant gene *Nrf2 *is reduced in *Bmal1KO *661W cells; although, presumably many transcript rhythms are affected by *Bmal1 *deletion. It is not clear that the *Nrf2 *rhythm is involved in the antioxidant (Figure 4B), GPx reductions (Figure 4C), or the cell viability of Figure 3. As the authors note, “Of the many antioxidants present in cells,” that *Nrf2 *and GPx are just two examples of antioxidant-related cellular components. I think Figure 4 works as a figure of three examples of reduced antioxidant-related measures, but it should be clear that these are not being presented as validated causes of the other phenotypes presented like circadian survival rate of Figure 3.

- Can the authors comment on the source of the *Bmal1 *cDNA that was used in the rescue? The Bmal1 rescue is under the control of the CMV promoter, not a circadian promoter, correct? This would yield a constitutive expression of Bmal1 itself for the rescue.  This may not change the results of the *Bmal1* rescue, but I think it is worth mentioning.
- In the discussion of the persistence of rhythms after deletion of *Bmal1*, the authors point to Figure 4B as an example of circadian variation. To be fair, this figure panel shows there is a difference at 2 points in both WT and BMKO cells, but not necessarily a circadian rhythm in antioxidant capacity.
- There are a couple of editing corrections:
  a) In the Western blot section of the Methods it says samples were heated to 950degC, instead of 95degC.
  b) In the discussion paragraph on the persistence of rhythms in *BmalKO *cells, I think the first sentence “in” should be “is”(…the antioxidant capacity of 661-BKO cells is the presence...).

Overall the paper shows a creative way to assess the rhythms of oxidative stress responses in cone-like cells.

Is the work clearly and accurately presented and does it cite the current literature?

Yes

If applicable, is the statistical analysis and its interpretation appropriate?

Yes

Are all the source data underlying the results available to ensure full reproducibility?

Yes

Is the study design appropriate and is the work technically sound?

Yes

Are the conclusions drawn adequately supported by the results?

Yes

Are sufficient details of methods and analysis provided to allow replication by others?

Yes

Reviewer Expertise:

retina, cornea, opsins, circadian rhythms

I confirm that I have read this submission and believe that I have an appropriate level of expertise to confirm that it is of an acceptable scientific standard.

# Response

**Published:** 2022-11-09

**Authors:** Kenkichi Baba

**Affiliations:** Morehouse School of Medicine, USA

Thank you Dr. Buhr for the valuable comments and suggestions.

*Comment: As the authors note, “Of the many antioxidants present in cells,” that Nrf2 and GPx are just two examples of antioxidant-related cellular components. I think Figure 4 works as a figure of three examples of reduced antioxidant-related measures, but it should be clear that these are not being presented as validated causes of the other phenotypes presented like circadian survival rate of Figure 3.*

 Response: We agree that there are several death pathways triggered by oxidative stress and what we observed in Figure 3 may not  exclusively caused by Nrf2 and GPX as we show in Figure 4. However, previous studies have reported that Nrf2 transcription is directly controlled by the circadian clock and Nrf2 is responsible for the control many of the genes mediating antioxidant response and thus we believe that our data suggest a direct involvement of Nrf2 in the response of 661W cells to an oxidative stress challenge. Similarly, we believe that the data show in Figure 4 on GPX activity provides strong evidence that GPX is a key player in the response observed in Figure 3 since this enzyme convert from H _2_O _2_ to H _2_O. We have also measured catalase activity, but unfortunately, catalase activity in 661W cells was found very low. Thus, we believe that the data presented in Figures 4-5 provide compelling evidence about the mechanism that is responsible for the data presented in Figure 3.

*Comment: Can the authors comment on the source of the Bmal1 cDNA that was used in the rescue? The Bmal1 rescue is under the control of the CMV promoter, not a circadian promoter, correct? This would yield a constitutive expression of Bmal1 itself for the rescue.  This may not change the results of the Bmal1 rescue, but I think it is worth mentioning.*

 Response: We have added the reference for the Bmal1 cDNA. Bmal1 expression is under the control of the CMV promoter, therefore we believe it would be expressed steadily in these cells.

*Comment: In the discussion of the persistence of rhythms after deletion of Bmal1, the authors point to Figure 4B as an example of circadian variation. To be fair, this figure panel shows there is a difference at 2 points in both WT and BMKO cells, but not necessarily a circadian rhythm in antioxidant capacity.*

 Response: We agree with the comment, and we have replaced the term circadian with “a time dependent change”

*Comment: a) In the Western blot section of the Methods it says samples were heated to 950degC, instead of 95degC.*

 Response: Thank you. We corrected it to 95 C.

*Comment: b) In the discussion paragraph on the persistence of rhythms in BmalKO cells, I think the first sentence “in” should be “is”(…the antioxidant capacity of 661-BKO cells is the presence...).*

 Response: Thank you for noticing this typo. We have corrected the text as suggested.
